# Agent-based simulation for weekend-extension strategies to mitigate influenza outbreaks

**DOI:** 10.1186/1471-2458-11-522

**Published:** 2011-06-30

**Authors:** Liang Mao

**Affiliations:** 1 Department of Geography, University of Florida, Gainesville, Florida, 32611, USA

## Abstract

**Background:**

Non-pharmaceutical strategies are vital in curtailing impacts of influenza and have been intensively studied in public health. However, few strategies have explicitly utilized the weekend effect, which has been widely reported to be capable of reducing influenza infections. This study aims to explore six weekend-extension strategies against seasonal and pandemic flu outbreaks.

**Methods:**

The weekend-extension strategies were designed to extend regular two-day weekend by one, two and three days, respectively, and in combination with either a continuous or discontinuous pattern. Their effectiveness was evaluated using an established agent-based spatially explicit simulation model in the urbanized area of Buffalo, NY, US.

**Results:**

If the extensions last more than two days, the weekend-extension strategies can remarkably reduce the overall disease attack rate of seasonal flu. Particularly, a three-day continuous extension is sufficient to suppress the epidemic and confine the spread of disease. For the pandemic flu, the weekend-extension strategies only produce a few mitigation effects until the extensions exceed three days. Sensitivity analysis indicated that a compliance level above 75% is necessary for the weekend-extension strategies to take effects.

**Conclusion:**

This research is the first attempt to incorporate the weekend effect into influenza mitigation strategies. The results suggest that appropriate extensions of the regular two-day weekend can be a potential measure to fight against influenza outbreaks, while minimizing interruptions on normal rhythms of socio-economy. The concept of weekend extension would be particularly useful if there were a lack of vaccine stockpiles, e.g., in countries with limited health resources, or in the case of unknown emerging infectious diseases.

## Background

Despite advances in medical sciences, influenza (commonly known as flu) remains a remarkable threat to the public health and socio-economy as a whole. Seasonal flu typically infects 10%~20% of the US population every year [1]. The pandemic H1N1 influenza (the 2009 swine flu) was recently reported to be responsible for 274,000 hospitalizations and 12,470 deaths in the US[2]. Due to the rapid mutation and swift spread of flu virus, preparedness for imminent pandemics is now a top priority of public health[3]. Among the core issues of preparedness is the study of mitigation strategies that can minimize impacts of influenza on human society.

Non-pharmaceutical mitigation strategies, such as the household quarantine, workplace/school closure, and travel restriction, had been embedded within the latest framework of influenza prevention and control recommended by the CDC's Advisory Committee on Immunization Practices [4]. These strategies are critical because they represent the only type of intervention measure guaranteed to be available against a novel strain of influenza in the early phases of a pandemic [5]. Their ultimate goals are to reduce infections and delay transmission, thereby allowing time to implement pharmaceutical strategies, such as vaccination and antiviral prophylaxis. Many studies, however, have pointed out that the non-pharmaceutical strategies are often difficult to put into practice, since their effectiveness is highly dependent on the compliance of population [6]. Furthermore, these strategies may infringe on human rights and involve psychological, ethical and legal issues, e.g., limiting free movement of individuals. A recent evaluation had concluded that there was a general lack of scientific evidence or expert consensus for implementing these strategies[7]. Due to these drawbacks, the exploration of non-pharmaceutical strategies remains an actively pursued topic in public health.

This paper proposes a new type of non-pharmaceutical strategies that extended the regular two-day weekend for the purposes of interrupting influenza transmission and mitigating disease impacts, referred to as 'weekend-extension strategies'. In the current literature, few mitigation strategies have considered the reductive effect of weekend on influenza transmission, although this effect has been widely reported [8-10]. For example, the study by Hens et al. in eight European countries estimated a 10~20% reduction in influenza infections during weekend when compared to weekdays [10]. Research by both Lee et al. and Cooley et al. attributes the variability of influenza incidence to the weekday-weekend effect [8,9]. A primary reason is that most workplaces and schools are closed simultaneously during weekend, and thus fewer human contacts take place as opposed to weekdays. For instance, a survey by McCaw et al. indicated that an individual has 2~4 more personal contacts during weekend than weekdays[11]. A study of university students by Edmunds et al. also found that individuals made 26 contacts per day during weekdays, but only 19 per day during weekend[12]. These studies imply that extending the weekend period might be an effective strategy to mitigate influenza outbreaks.

To test the effectiveness of weekend-extension strategies, an established agent-based spatially-explicit model was developed for the urbanized area of Buffalo, New York, US. The model simulated these strategies and produced epidemic outcomes for evaluation. The remainder of this article is organized into following sections. The second section introduces the study area and methods, including the design of weekend-extension strategies and the agent-based simulation model. The third section presents simulation results and compares the effectiveness between strategies. The fourth section discusses model outcomes and implications, and the final section concludes the article.

## Methods

### Design of weekend-extension strategies

Two dimensions were involved in the design of weekend-extension strategies. One dimension concerned the length of extensions, i.e., how long the regular Saturday and Sunday weekend should be extended. The other dimension specified the pattern of extensions, i.e., in which manner the extensions should be applied. A continuous pattern means that the additional weekend follows right after the regular weekend and lasts continuously, e.g., Saturday + Sunday+ (Monday +Tuesday). A discontinuous pattern separately arranges the additional weekend within a week, for instance, Saturday + Sunday + (Tuesday +Thursday). The continuous extensions produce longer and less frequent interruptions on influenza transmission, while the discontinuous extensions cause shorter and more frequent interruptions. In this research, three lengths of extensions were investigated, including one, two and three days, in combination with the two patterns of extensions (continuous and discontinuous). The combination of the two dimensions (3 lengths × 2 patterns) resulted in six strategies to be evaluated (Table 1). During the additional weekend, all businesses (including schools) were closed except for service-oriented places, such as utility companies, health facilities, restaurants, and grocery stores. Individuals were assumed to follow activity patterns of either Saturday or Sunday, e.g., staying at home, visiting service places, or meeting with friends in neighbor households.

**Table 1:** Design of six weekend-extension strategies and their abbreviations

Patterns Lengths (day)	Continuous	Discontinuous
One	Monday (Mon)	Wednesday (Wed)
Two	Monday + Tuesday (Mon + Tue)	Tuesday + Thursday (Tue + Thur)
Three	Monday +Tuesday + Wednesday (Mon+Tue+Wed)	Monday + Wednesday + Friday (Mon+Wed+Fri)

### Study area and data collection

The six proposed strategies were investigated in the urbanized area of Buffalo, New York, US. This study area was chosen because an influenza simulation model had been previously established [13]. The input data included the information about 985,001 individuals and 400,870 households from US Census 2000 [14], as well as a database of 36,839 business locations in year 2009 [15]. Individuals were assumed to travel through a local transportation system, take their daily activities at homes and business locations, have contact with one another, and expose themselves to influenza infection.

### Agent-based influenza model

The established agent-based model first simulated a contact network in the study area, which provided a basis for influenza transmission [13]. The network consists of 985,001 discrete individuals as nodes and their daily contacts as links. Individual contacts were assumed to take place during three time periods in a day and at four types of locations. The three time periods included: daytime, pastime and nighttime, while the four types of locations referred to homes, workplaces (schools, financial offices, administrative units, industrial factories, etc.), service places (utility companies, health facilities, grocery stores, etc.), and neighbor households (households in the same census block group). Modeled individuals traveled between time and location, and had contact with different groups of individuals, such as family members, co-workers, clerks, and friends. These spatiotemporally varying contacts linked all individuals into a city-wide network.

To construct such a contact network, three populations of daytime, nighttime and pastime were synthesized respectively and then linked together [13]. The census data was used to build the nighttime population of individuals and households that matched the real age and household structures. The nighttime population was assigned to business locations to create the daytime population, according to the travel time to work and industrial types of business locations. Next, the pastime population was generated based on the information of previous two populations and a regional travel survey [16]. Under the constraints of travel statistics, individuals were further allocated to service places and neighbor households. Detailed algorithms to generate these three populations can be referred to the work by Mao and Bian [13].

**Figure 1:**
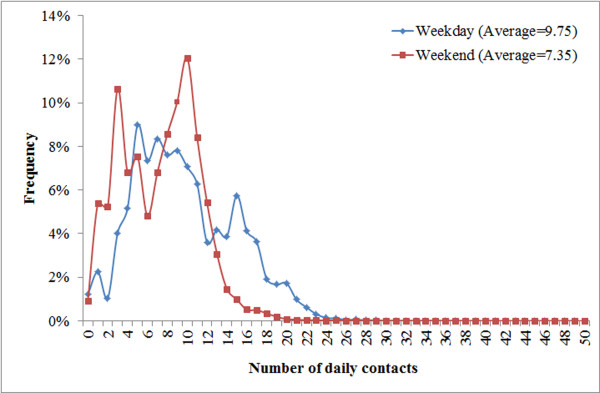
**Contact patterns during a weekday and a weekend**. Frequency distributions of individual contacts during a regular weekday (blue) and a weekend (red). The *X *axis represents the number of daily contacts, and the *Y *axis denotes the corresponding frequency in the population.

This research further distinguished the weekday and weekend activities of individuals based on the regional travel survey. Specifically, individuals were not assigned to work during weekend (both regular and additional) except for those who work at service places. The trips to workplaces or schools (for children under 18 years old) were replaced by either staying at homes, or visiting service places/neighbor households. The simulated individual contacts during a weekday and a weekend day (Figure 1) have clear power-law distributions, consistent with the observed "scale-free" property of human social networks. The average number of contacts at the weekend is 2.4 fewer than that in the weekday, because fewer workplace contacts happen at the weekend.

### Flu infectivity scenarios

To initialize influenza transmission through the modeled network, five infectious individuals were randomly seeded into the study area at the first day of simulation. The simulation took a tri-daily time step and lasted for 200 days. During each time step, an individual held one of four infection status, i.e., susceptible, latent, infectious, or recovered. The infection proceeded for 7-10 days, with 2 days latent, 1 day asymptomatic and infectious, followed by 4-7 days infectious varying over age groups (Children: <16 years; Adults: 16-64 years; Senior: >64 years)[17]. The probability of developing symptoms once infected was set to 0.5 [18], and symptomatic individuals were referred to as 'influenza cases' in subsequent discussion. Further, a proportion of symptomatic individuals was selected to withdraw to home based on previous surveys of flu self-care behavior [19,20]. The stochastic simulation randomized the five infective seeds, the daily contacts of individuals and the infections through contacts, as well as the development of symptoms and the withdrawal-to-home of symptomatic individuals.

The transmission of influenza was simulated by repeatedly tracing susceptible contacts of infectious individuals, and identifying who will receive the infection in the next time step. The receipt of infection was modeled as a stochastic event determined by the age groups of receivers and the infectivity of viral strains. The viral infectivity was specified by *R*_*0 *_(the basic productive number), which is defined as the number of secondary cases caused by a single infected case in a wholly susceptible population[21]. Relevant to this research, two scenarios of flu infectivity were established to examine the effectiveness of weekend-extension strategies: a seasonal flu scenario (*R*_*0 *_= 1.4) and a pandemic flu scenario (*R*_*0 *_= 2.0) [22]. Compared to the seasonal flu, the pandemic flu occurs rarely but transmits more easily between human beings and spreads quickly all over the world, because the virus is often novel to human immune system. Under each flu scenario, a baseline outbreak and the six strategies (in Table 1) were simulated and compared with one another. The baseline outbreak represented a 'no response' situation with no strategies applied, and served as a reference for comparison purposes. To validate the model, the baseline outcomes were compared to CDC weekly reports of laboratory confirmed specimens in 2004-05 flu epidemic in the study area [23].

### Measures of control effectiveness

The six weekend-extension strategies were assumed to be implemented when the cumulative number of influenza cases exceeded 1,000 (about 1‰ of the population). The simulation results recorded the time period and location of every infection event. The results were aggregated into a daily epidemic curve, which depicted the number of new influenza cases per day during the course of an epidemic. Characteristics associated with the epidemic curve were also extracted, including an overall attack rate (the total percent of influenza cases in the population), an epidemic peak, and peak time. An outbreak was deemed to be successfully controlled if the overall attack rate can be reduced below 10% [5]. In addition, the attack rate <5% was used as a criterion for evaluating if an outbreak can be further prevented, since reported flu epidemics often have an attack rate of 5% or higher[1]. To compare strategies, a relative effectiveness was calculated as a ratio of the attack rate reduced by a strategy to the baseline attack rate. This relative effectiveness ranges from 0 to 1, with 0 indicating no effectiveness on influenza outbreaks. Further, a sensitivity analysis and ANOVA test were conducted upon a range of compliance levels, including 100%, 90%, 75%, 50% and 0% of businesses, accounting for the situation that business owners were reluctant to suspend their businesses.

This research also displayed the spatial effectiveness of mitigation from each strategy. The simulation results were converted from points of infections to grid cells of infections per km^2^, as infection intensity maps. A kernel density function was utilized to interpolate the total number of infections at every cell location during the 200-day simulation. The cell size was set to 50 × 50 m because it approximated the average extent of land parcels in the study area. An effective strategy was expected to reduce the infection intensity at every location and confine the spatial extent of affected areas. Both the epidemic curves and infection intensity maps showed the mean of 50 model runs to average out the variation caused by the stochastic simulation.

## Results

### Baseline outbreaks of seasonal and pandemic flu

**Figure 2:**
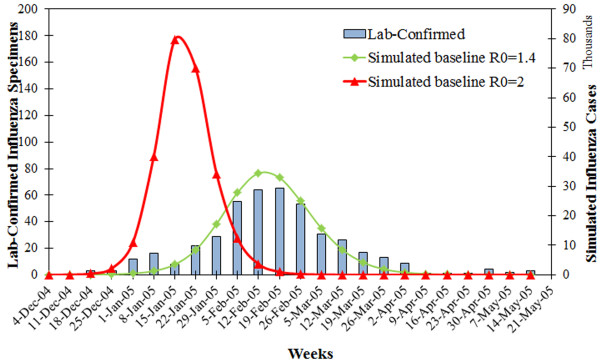
**Baseline scenarios of seasonal and pandemic flu**. Simulated baseline outbreaks of influenza epidemic (Green) and pandemic (Red), compared to the reported influenza epidemic by CDC in the same area for year 2004-5 (Blue bars). The bars represent the number of confirmed influenza specimens per week during the epidemic 2004-5(Left *Y *axis). The curves represent newly influenza cases per week during the course of an epidemic or a pandemic (Right *Y *axis).

The modeled epidemic curve under *R*_*0 *_= 1.4 matched well with the observed time course of a seasonal flu outbreak in year 2004-05 (Figure 2), although the magnitude of simulated curve was larger than the reported. This is because a large proportion of infected people may not manifest symptoms or seek health care, and thus the number of influenza cases was highly under-reported by CDC. In this sense, the simulation model performed well in predicting the time trend, and at least allowed a representation of the worse case result. The pandemic flu (*R*_*0 *_= 2.0) caused an earlier and higher epidemic peak with more influenza cases, because this virus strain is far more contagious and spreads faster.

### Continuous weekend-extension strategies

**Figure 3:**
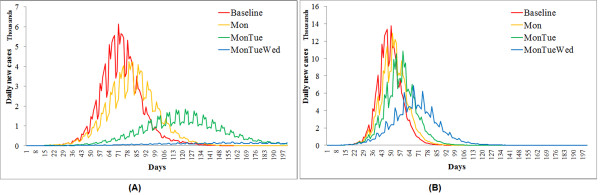
**Continuous weekend-extension strategies (1)**. Simulated epidemic curves from three continuous weekend-extension strategies, in comparison with the baseline epidemic. *Y *axis indicates the number of newly influenza cases per day during the 200 day simulation. All curves are averages of 50 randomly seeded simulation runs.

In the case of seasonal flu, the effectiveness of three continuous strategies differ statistically from one another (*F *= 6.89 and *p*-value = 0.02 from ANOVA). Extending the weekend to Monday mildly reduced the overall attack rate from 18.61% to 16.14% (Table 2) and slightly delayed the peak time by one week (Figure 3a). Apparent mitigation effects occurred when the weekend was extended by two consecutive days or longer. Given a high compliance level (>90%), the 'Mon+Tue' strategy can lessen the overall attack rate close to 10% and postpone the peak time by almost 7 weeks. Extending the weekend by three consecutive days (Mon+Tue+Wed) was capable of controlling the attack rate under 10%, given a compliance level above 75%. Provided a full compliance, the 'Mon+Tue+Wed' strategy can even prevent the epidemic by curtailing the attack rate far below 5%. Turning to the pandemic flu scenario (Figure 3b), the three continuous weekend-extension strategies did not perform as effectively as in the seasonal flu scenario, but their effectiveness remained statistically different (*F *= 14.7 and *p*-value = 0.001). Extending the weekend by one or two days produced a few effects on the overall attack rate and peak time. Only the three-day continuous extension can significantly lower the overall attack rate from 26% to 20% (Table 2). All three strategies were not capable of controlling the pandemic flu independently.

**Table 2:** Sensitivity of continuous weekend-extension strategies to compliance levels

Effectiveness		***R***_***0 ***_**= 1.4**		***R***_***0 ***_**= 2.0**	
		
Compliance		**Overall attack rates % (95% CI**^**a**^**)**	**Relative effectiveness**^**b**^	Overall attack rates % (95% CI)	Relative effectiveness
Baseline		18.61 (18.54, 18.71)	0.00	26.43 (26.36, 26.53)	0.00

Mon	100%	16.14 (16.03, 16.26)	0.13	25.52 (25.43, 25.62)	0.03
	90%	16.41 (16.28, 16.54)	0.12	25.58 (25.49, 25.67)	0.03
	75%	16.83 (16.74, 16.96)	0.10	25.74 (25.67, 25.82)	0.03
	50%	17.53 (17.39, 17.65)	0.06	26.01 (25.95, 26.09)	0.02

Mon+Tue	100%	11.82 (11.58, 12.11)	0.36	23.84 (23.73, 23.94)	0.10
	90%	12.98 (12.80, 13.17)	0.30	24.10 (24.00, 24.22)	0.10
	75%	14.23 (13.93, 14.46)	0.23	24.55 (24.41, 24.96)	0.07
	50%	15.99 (15.81, 16.22)	0.14	25.28 (25.16, 25.36)	0.04

Mon+Tue	100%	3.26 (1.47, 4.79)	0.82	20.30 (20.16, 20.42)	0.23
+Wed	90%	7.24 (4.87, 8.21)	0.61	21.15 (21.01, 21.34)	0.20
	75%	10.92 (10.27, 11.37)	0.41	22.31 (22.17, 22.43)	0.16
	50%	14.45 (14.13, 14.80)	0.22	24.00 (23.81, 24.15)	0.10

^a^95%CI stands for the 95% confidence intervals from 50 model realizations.

^b^Relative effectiveness = (Baseline attack rate -Strategy-specific attack rate)/Baseline attack rate.

Table 2 compares the overall attack rates and relative effectiveness resulted from three continuous weekend-extension strategies (Mon, Mon+Tue, and Mon+Tue+Wed). Sensitivity analysis was conducted on each strategy under five compliance levels of 0%, 50%, 75%, 90%, and 100%.

The compliance levels of businesses had profound effects on the control effectiveness (Table 2). The longer the extension of weekend, the lower the compliance level was needed to achieve the same control effectiveness. For the seasonal flu, increasing the compliance level from 50% to 90% can improve the control effectiveness by 2-3 times (*F *= 4.40, *p*-value = 0.008 from ANOVA). With respect to pandemic flu, the compliance levels caused less variation in control effectiveness but remain statistically different (*F *= 3.36 and *p*-value = 0.02). Even the 100% compliance cannot help any of the three strategies to dampen the flu pandemics.

**Figure 4:**
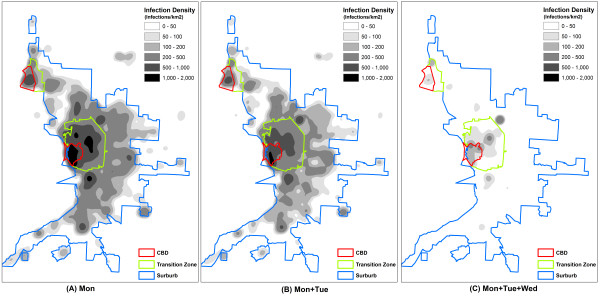
**Continuous weekend-extension strategies (2)**. Spatial patterns of infections resulted from the three continuous weekend-extension strategies, given *R*_*0 *_= 1.4 and compliance level = 100%: (a) Mon, (b) Mon+Tue, and (c) Mon+Tue+Wed. Each 50 m × 50 m cell value indicates the total number of infections during the 200 days at the cell location (infections/per km^2^). All cell values are averages of 50 randomly seeded simulation runs.

The spatial effectiveness against seasonal flu was of interest as well. For the ease of description, the intensity maps divided the study area into three zones: a central business district (CBD), a transition zone, and a suburb (Figure 4). The zonal delineation was based on densities of households and businesses, as well as the layout of major roads. The 'Mon' strategy (Figure 4a) failed to prevent the wide dispersion of influenza. The entire study area was dominated by high intensity of infections (>200 infections per km^2^). The 'Mon+Tue' strategy (Figure 4b) apparently mitigated infections in all three zones, but could not downsize the affected areas. The 'Mon+Tue+Wed' strategy had an outstanding effect on the spatial dispersion of influenza (Figure 4c). The affected areas were largely reduced, leaving the extensive suburb with only a small number of infection clusters.

### Discontinuous weekend-extension strategies

**Figure 5:**
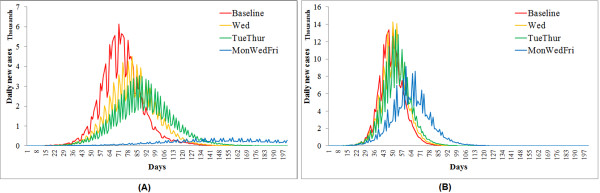
**Discontinuous weekend-extension strategies (1)**. Simulated epidemic curves from the three discontinuous weekend-extension strategies, in comparison with the baseline epidemic. *Y *axis indicates the number of newly influenza cases per day during the 200 day simulation. All curves are averages of 50 randomly seeded simulation runs.

Compared to the continuous strategies, the discontinuous strategies resulted in epidemic curves with more oscillations (Figure 5). This is because the days on and off work switched more frequently and posed more interruptions on flu transmission. The ANOVA indicated significant differences between the effectiveness of three strategies: *F *= 3.96 and *p*-value = 0.05, given *R*_*0 *_= 1.4; *F *= 5.62 and *p*-value = 0.02, given *R*_*0 *_= 2.0. The sensitivity analysis showed increased control effectiveness with a longer weekend period and a higher compliance level of businesses (Table 3). Only the three-day discontinuous extension ('Mon+Wed+Fri') with a 90% compliance level can control the seasonal flu (attack rate <10%). If the compliance could be raised to 100%, the three-day extension would be adequate to prevent the seasonal flu epidemic (attack rate <5%). For the pandemic flu, however, none of these three discontinuous strategies produced significant mitigation effects. Even the three-day extension strategy with a 100% compliance level failed to reduce the attack rate below 20%.

**Table 3:** Sensitivity of discontinuous weekend-extension strategies to compliance levels

Effectiveness		***R***_***0 ***_**= 1.4**		***R***_***0 ***_**= 2.0**	
		
Compliance		**Overall attack rates % (95%CI**^**a**^**)**	**Relative effectiveness**^**b**^	Overall attack rates % (95%CI)	Relative effectiveness
Baseline		18.61 (18.54, 18.71)	0.00	26.43 (26.36, 26.53)	0.00

Wed	100%	16.53 (16.42, 16.64)	0.11	25.67 (25.58, 25.75)	0.03
	90%	16.76 (16.60, 16.90)	0.10	25.73 (25.67, 25.79)	0.03
	75%	17.10 (16.98, 17.21)	0.08	25.86 (25.77, 25.95)	0.02
	50%	17.70 (17.54, 17.87)	0.05	26.08 (26.01, 26.14)	0.01

Tue+Thur	100%	14.43 (14.30, 14.58)	0.22	24.77 (24.68, 24.84)	0.06
	90%	15.00 (14.87, 15.16)	0.19	24.93 (24.83, 25.00)	0.06
	75%	15.73 (15.63, 15.83)	0.15	25.23 (25.17, 25.29)	0.05
	50%	16.90 (16.79, 17.02)	0.09	25.70 (25.62, 25.77)	0.03

Mon+Wed+	100%	5.49 (1.59, 6.88)	0.69	21.41 (21.27, 21.52)	0.19
Fri	90%	9.23 (8.11, 9.80)	0.50	22.07 (21.95, 22.23)	0.16
	75%	11.92 (11.64, 12.27)	0.36	23.00 (22.81, 23.11)	0.13
	50%	14.95 (14.72, 15.28)	0.20	24.42 (24.32, 24.56)	0.08

^a^95%CI stands for the 95% confidence intervals from 50 model realizations.

^b^Relative effectiveness = (Baseline attack rate -Strategy-specific attack rate)/Baseline attack rate.

Table 3 compares the overall attack rates and relative effectiveness resulted from three discontinuous weekend-extension strategies (Wed, Tue+Thur, and Mon+Wed+Fri). Sensitivity analysis was conducted on each strategy under five compliance levels of 0%, 50%, 75%, 90%, and 100%.

**Figure 6:**
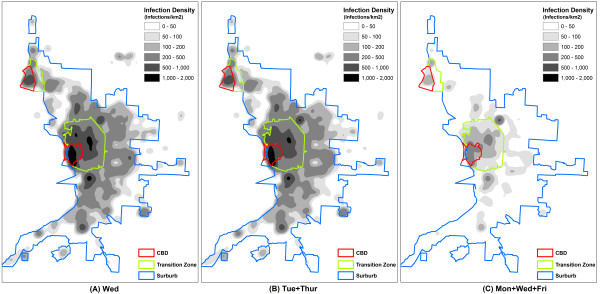
**Discontinuous weekend-extension strategies (2)**. Spatial patterns of infections resulted from the three discontinuous weekend-extension strategies given *R*_*0 *_= 1.4 and compliance level = 100%: (a) Wed, (b) Tue+Thur, and (c) Mon+Wed+Fri. Each 50 m × 50 m cell value indicates the total number of infections during the 200 days at the cell location (infections/per km^2^). All cell values are averages of 50 randomly seeded simulation runs.

From a spatial perspective, the 'Wed' strategy (Figure 6a) contributed little to containing the extensive spread of seasonal flu. A majority of the study area underwent a high intensity above 200 infections per km^2^. The 'Tue+Thur' strategy (Figure 6b) slightly changed the spatial patterns and produced small improvement as opposed to the 'Wed' extension strategy. The 'Mon+Wed+Fri' strategy (Figure 6c) eliminated infections in most parts of the suburb, but the CBD and transition zone remained at a moderate intensity of around 100 infections per km^2^. Geographically, this strategy failed to isolate the affected area into small clusters of infections.

### Shares of infections by location

**Figure 7:**
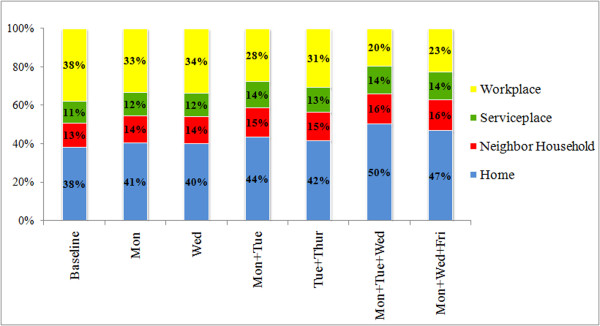
**Share of Infections by location**. Share of infections (in percentage) at the four types of locations respectively, as a result of the six weekend-extension strategies under *R*_0 _= 1.4. All percentages are averages of 50 randomized model runs.

It is also noteworthy to compare how these weekend-extension strategies move the foci of infections between the four types of location, i.e., home, workplace, service place, and neighbor household (Figure 7). For each strategy, the resultant shares of infections (%) were calculated by counting the number of infections occurring at each type of location, and dividing it by the total number of infections during the epidemic. For the baseline scenario (*R*_*0 *_= 1.4), the workplaces and homes had the same share of infections (38%). As the extension days increase up to 3 days, the weekend-extension strategies heavily reduced the share of infections at workplaces from 38% to 20%, while greatly elevated the share at homes from 38% to 50%. The shares of infections at neighbor households and service places were also slightly enlarged by 3%.

## Discussion

Analyses above imply that the effectiveness of weekend-extension strategies is sensitive to the length of extensions, the compliance level of businesses, and the infectivity of influenza virus. The three-day extension strategy is capable of controlling seasonal flu epidemics, and even prevents the epidemics if a high compliance level can be achieved. The reason is that individuals would have far fewer contacts due to a largely reduced weekday schedule. Most infections are limited within households, but cannot spread out to workplaces until individuals go back to work, making the transmission inefficient. However, the weekend-extension strategies alone are not able to control pandemic flu, because this virus strain is so contagious that only a few human contacts could sustain the chain of transmission. To be effective in flu pandemics, these strategies need to be complemented by other pharmaceutical strategies that offer direct protection to individuals, such as the mass vaccination and antiviral prophylaxis.

Comparison showed that the three discontinuous extension strategies (Table 3) are less effective than their continuous counterparts (Table 2), because the resultant attack rates are 1~3% higher. This suggests that longer and less frequent interruptions on influenza transmission would be more effective in disease control than shorter and more frequent interruptions. A probable explanation is that the transmission of influenza would be doubly effective if infectious individuals constantly meet with susceptible individuals at both homes and workplaces. A continuous weekend extension allows disease transmission between household members, but eliminates the transmission among co-workers for a relative long period (e.g., 3~5 days). Hence, the possible routes for transmission are quickly exhausted at homes, and epidemics cannot further develop until individuals go back to work. In contrast, a discontinuous weekend extension allows influenza not only spreading within households, but also transmitting intermittently within workplace contexts (e.g., every other day). The pool of susceptible individuals can be replenished at a short time interval, resulting in more infections.

As people spend more time at home during the extended weekend, it is not surprising that the foci of infection gradually move from workplaces to homes (Figure 7). This also explains why the highest intensity of infections often occurs within the CBD (Figure 4 and 6). With a household density of 1,189.30/km^2^, the CBD has a much higher concentration of residents than the transition zone (879.30/km^2^) and suburb (21.30/km^2^). The dense contact network woven by concentrated residents retained the CBD relatively insensitive to the weekend-extension strategies. In this case, the CBD could be further targeted by household prophylaxis or household quarantine strategies, as complementary interventions. A vaccination program prioritizing CBD residents would also be a wise preparation for weekend-extension strategies.

In addition to the land use patterns, the travel behavior of individuals is another key factor for disease dispersion [13]. This explains why the three-day continuous extension strategy is the most effective to confine the spatial spread of seasonal flu. The long weekend period greatly reduces the travel between homes and workplaces, which is a major component of individual daily activities. Many infectious individuals stay home for 5 consecutive days, "using up" the infectious period of influenza virus. When these individuals go back to work, they are no longer infectious and cannot infect their co-workers, thereby limiting the long-distance dispersion of influenza.

From perspectives of psychology, ethics and law, the weekend-extension strategy may involve milder issues than other non-pharmaceutical strategies, such as the case isolation or household quarantine. It has been widely reported that many non-pharmaceutical strategies, particularly for long duration, can cause loneliness, emotional detachment and infringement of individual rights, such as the freedom of movement [24]. Differently, the weekend-extension strategy only causes short-term social separation, and allows people to move freely to anywhere they want during the extended weekend. Many ethical and legal issues therefore could be possibly mitigated or avoided.

The socio-economic loss from work/school absenteeism is a potential problem for implementing weekend extension strategies. One solution is to encourage people to work at home during the extended weekend, complete business transactions through telecommunication, and take courses online. In such a manner, the face-to-face contacts for infection are reduced, while long-term interruptions on socio-economy could be minimized. If the long-distance working and learning are not feasible for certain occupations, an alternative is to grant these groups of people a higher priority for receiving pharmaceutical interventions.

## Conclusions

This research is the first attempt to consider the weekend effect on influenza control and prevention, and starts a new direction for designing mitigation strategies. The effectiveness of weekend-extension strategies depends on the length and pattern of extensions, as well as the compliance of businesses. The simulation results suggest that the extension of regular weekend by more than two days can significantly mitigate seasonal flu epidemics. For pandemic flu, the weekend-extension strategies are not effective alone, but would be useful complements to pharmaceutical strategies.

Like other non-pharmaceutical strategies, the weekend-extension strategy could be a feasible measure for countries with limited health resources, because no stockpiles of vaccines and antiviral drugs are needed. Although influenza is taken as an example in this research, it is believed that the concept of weekend extensions can also help fight other emerging infectious diseases that are poorly understood and unprepared for, such as new strains of influenza, SARS (Severe acute respiratory syndrome), and Ebola. With the advance in telecommunication technologies and the shift of working styles from workplace to home, the weekend-extension strategy may have long-term containment benefits for this class of diseases, and would be a wise option for public health planners in the near future.

## Competing interests

The author declares no competing interests.

## Authors' contributions

LM conceived and designed the work, performed all coding and simulation, carried out all analyses, and is the author of the manuscript.

## Pre-publication history

The pre-publication history for this paper can be accessed here:

http://www.biomedcentral.com/1471-2458/11/522/prepub
